# Associations between body composition, hydration status, and sleep architecture in obstructive sleep apnea

**DOI:** 10.3389/fendo.2025.1666026

**Published:** 2025-11-10

**Authors:** Ya-Min Chuang, Wen-Te Liu, Cheng-Yu Tsai, Yi-Chih Lin, Kin Fai Ho, Kai-Jen Chuang, Hsiao-Chi Chuang

**Affiliations:** 1 Graduate Institute of Medical Sciences, College of Medicine, Taipei Medical University, Taipei, Taiwan; 2 Sleep Center, Shuang Ho Hospital, Taipei Medical University, New Taipei City, Taiwan; 3 School of Respiratory Therapy, College of Medicine, Taipei Medical University, Taipei, Taiwan; 4 Division of Pulmonary Medicine, Department of Internal Medicine, Shuang Ho Hospital, Taipei Medical University, New Taipei City, Taiwan; 5 School of Biomedical Engineering, College of Biomedical Engineering, Taipei Medical University, New Taipei City, Taiwan; 6 Department of Otolaryngology, School of Medicine, College of Medicine, Taipei Medical University, Taipei, Taiwan; 7 Jockey Club School of Public Health and Primary Care, The Chinese University of Hong Kong, Hong Kong, Hong Kong SAR, China; 8 Key Laboratory of Aerosol Chemistry & Physics, Institute of Earth Environment, Chinese Academy of Sciences, Xi’an, China; 9 School of Public Health, College of Public Health, Taipei Medical University, New Taipei City, Taiwan; 10 Department of Public Health, School of Medicine, College of Medicine, Taipei Medical University, Taipei, Taiwan; 11 Cell Physiology and Molecular Image Research Center, Wan Fang Hospital, Taipei Medical University, Taipei, Taiwan

**Keywords:** AHI, body composition, hydration status, OSA, polysomnography, sleep architecture

## Abstract

**Introduction:**

Obstructive sleep apnea (OSA) is linked to increased cardiovascular and metabolic risks. Although body mass index is a recognized risk factor, the roles of fat mass, muscle mass, and hydration status in influencing OSA severity and sleep architecture remain unclear. This study examined the associations between body composition and apnea-hypopnea index, sleep efficiency, and sleep stages across sex, age, and BMI strata.

**Materials and methods:**

In this cross-sectional study, data from 5,381 adults undergoing overnight polysomnography and bioimpedance analysis were analyzed. Multivariable linear regression, adjusted for age, sex, and BMI were used, with additional analyses stratified by sex, age, and BMI.

**Results:**

A unit increase in fat percentage (FATP) and fat mass (FATM) were associated with increasing AHI, whereas increase in bone mass, and FFM were associated with decreasing AHI, particularly in younger adults. Intracellular and total body water associated with increasing AHI. Stratified analyses revealed stronger associations in females and individuals with obesity. A unit increase in adiposity associated with increasing WASO, while bone mass (BONEM) reduced WASO. A unit increase in BMI and water content were linked to increasing N1 stage, whereas increase in PMM and BONEM associated with increasing deeper stages. Age- and sex-specific analyses showed stronger adiposity-AHI associations in younger adults, while these including hydration parameters, tend to attenuate with age.

**Conclusion:**

Body composition and hydration status were significantly associated with sleep architecture and AHI. These findings support a personalized approach to sleep health and OSA management, emphasizing not only weight control but also muscle preservation and fluid balance optimization.

## 1 Introduction

Obstructive sleep apnea (OSA) is a common sleep disorder marked by recurrent upper airway collapse during sleep, leading to intermittent oxygen desaturation, disrupted sleep continuity and reduced overall sleep quality. OSA is associated with increased risks of cardiovascular disease, metabolic dysfunction, and neurocognitive decline (1). The apnea-hypopnea index (AHI), which quantifies respiratory events per hour of sleep, is widely used to assess OSA severity. Although obesity has been recognized as a major risk factor for OSA, growing evidence suggests that a more nuanced evaluation of body composition. Body composition (i.e., fat mass (FATM), muscle mass (PMM), bone mass (ONEM), and hydration status) may associate with the pathophysiology and severity of OSA.

Body composition, defined as the relative proportions of fat, muscle, bone, and body water, offers greater specificity than body mass index (BMI) in predicting health outcomes (2–4). Excess adiposity, particularly central and visceral fat, can exacerbate airway obstruction and respiratory instability during sleep (5–8), while greater muscle and bone mass may help preserve upper airway patency (9). Hydration parameters (i.e., total body water (TBW), extracellular water (ECW), and intracellular water (ICW)) may further influence tissue compliance and airway resistance; however, they remain insufficiently studied in the context of OSA.

Despite the well-established association between obesity and OSA (10, 11), limited research has explored specific components of body composition regulates sleep stage architecture. Sleep stages, including light sleep (N1), intermediate (N2), deep sleep (N3), and rapid eye movement (REM) sleep, are increasingly recognized as physiological markers of metabolic and restorative processes. Lighter sleep stages may reflect poor sleep quality and are associated with adverse metabolic outcomes, while deeper sleep stages support tissue recovery and energy balance (12). Together, the effects of fat distribution, muscle mass, and hydration on sleep stages remain unclear.

Sleep disturbances also represent a growing global health concern, with an estimated prevalence of 27% worldwide (13–16). In Taiwan, high rates of sleep dysfunction have been reported across various populations, often coexisting with lifestyle-related and chronic health conditions (17–19). Given the pervasive burden of sleep disorders and their systemic consequences, identifying modifiable physiological contributors such as body composition and fluid balance is essential for effective intervention. The objective of this study is to examine the effects of body composition on AHI, sleep efficiency (SE), and the architecture of sleep stages by sex, age, and BMI strata. The findings may help guide targeted physiology-informed interventions that extend beyond weight management to include strategies focused on muscle preservation, fluid regulation, and sleep stage optimization.

## 2 Materials and methods

### 2.1 Study population

This cross-sectional study enrolled 5,381 adult participants (aged 18–80 years) from a sleep center in New Taipei City, Taiwan. The inclusion criteria were subjects ages 18~80 years and diagnosed with OSA (overall AHI ≥ 5 events/hour) by a full-night polysomnography (PSG). We excluded patients with cardiovascular diseases, heart failure, chronic obstructive pneumonia disease, diabetes mellitus, venous insufficiency, renal failure, and patients with hemodialysis. The comorbidity was defined based on clinical diagnosis. The study protocol was reviewed and approved by the Joint Institutional Review Board of Taipei Medical University (TMU-JIRB No. N201912095). As this study employed a retrospective design and involved only routine clinical procedures, the requirement for informed consent was waived.

### 2.2 Body composition measurements

Body composition was measured before and after overnight PSG using a validated bioelectrical impedance analysis (BIA) device. Parameters recorded included fat mass (FATM), fat percentage (FATP), visceral fat level (VFATL), muscle mass (PMM), fat-free mass (FFM), bone mass (BONEM), and hydration compartments: total body water (TBW), extracellular water (ECW), and intracellular water (ICW). Participants were instructed to fast for 3–4 hours, avoid physical activity for at least 12 hours, and maintain adequate hydration before testing. Bladders were emptied prior to each measurement, and no food or drink was allowed between the pre- and post-sleep assessments.

### 2.3 Sleep parameter assessment

Sleep parameters were assessed using a digital PSG system (Embla N7000, Medcare, Reykjavik, Iceland), which recorded nasal and oral airflow, thoracic and abdominal respiratory effort, oxygen saturation (SpO_2_), body movement, sleeping position, and snoring events. All recordings were scored by certified sleep technologists in accordance with the American Academy of Sleep Medicine (AASM) criteria (20, 21). Apnea was defined as a ≥90% reduction in airflow lasting at least 10 seconds, with or without associated desaturation or arousal. Hypopnea was defined as a ≥30% but <90% reduction in airflow lasting at least 10 seconds, accompanied by either ≥3% oxygen desaturation or an arousal. The apnea-hypopnea index (AHI) was computed by dividing the total number of apneas and hypopneas events per hour by the total sleep time (TST) in hours. Sleep architecture was determined by calculating the percentage of TST spent in non-rapid eye movement sleep (N1, N2, N3) and rapid eye movement (REM) sleep. SE was defined as the ratio of TST to time in bed, expressed as a percentage. Wake after sleep onset (WASO) was measured in minutes as the cumulative duration of wakefulness after initial sleep onset.

### 2.4 Statistical analysis

All statistical analyses were performed using SPSS version 22.0.0.0 for Windows (SPSS Inc., Chicago, IL, USA). Data were first assessed for normality, and extreme values beyond the 1st and 99th percentiles were winsorized to limit the influence of outliers (22). Paired *t*-tests were applied to compare pre- and post-sleep values of body composition and metabolic parameters. Linear regression analyses were conducted using delta (Δ) values, defined as the difference between post- and pre-sleep measurements. These models examined associations with AHI and other sleep metrics, stratified by sex and body mass index (BMI) categories: normal (<24 kg/m²), overweight (24–27 kg/m²), and obese (≥27 kg/m²). Model 1 adjusted for age and sex, while Model 2 included additional adjustment for BMI. All models controlled for relevant covariates, including waist circumference, neck circumference, FATM, FATP, PMM, VFATL, TBW, ECW, ICW, BONEM, FFM, basal metabolic rate (BMR), metabolic age (METAAGE), impedance (IMP), phase angle (PHASEANGLE), and physical rating (PHYSRATE). Additional regression models were conducted to assess the associations of SE and WASO with body composition and physiological variables, with SE stratified into three categories (≥85%, 70–85%, ≤70%), adjusted for age and sex. Further models examined associations between body composition and sleep stages (N1, N2, N3, REM), adjusted for age and sex. Subgroup analyses included stratification by age group (<50 vs. ≥50 years), adjusted for sex and further stratification by both age and BMI for male and female participants. Regression results were presented as beta coefficients (β) with 95% confidence intervals (CI), and statistical significance was defined as *p* < 0.05.

## 3 Results

### 3.1 Characteristic of study subjects

**Figure 1:**
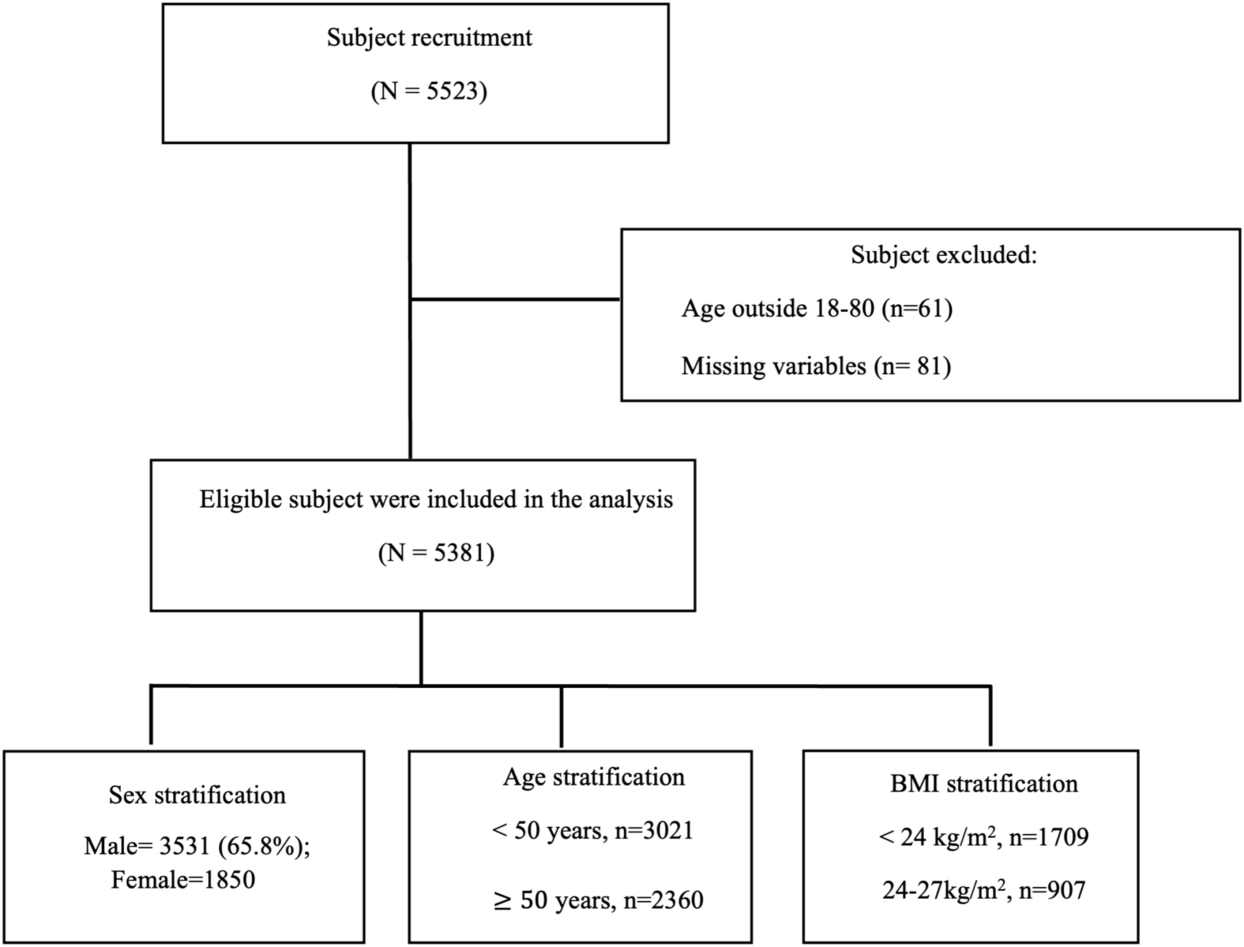
Flow diagram of participant recruitment, exclusion and stratification.

The study subject recruited in this study is shown in **Figure 1**. The characteristics of the 5,381 study participants are summarized in **Table 1**. The mean age was 47.17 ± 13.77 years, and 65.8% (n = 3,531) were male. The average BMI was 26.80 ± 5.10 kg/m². The mean neck and waist circumferences were 37.56 ± 4.70 cm and 91.67 ± 12.99 cm, respectively. The average TST was 4.49 ± 1.03 hours, and SE was notably low at 73.31 ± 16.64%. Participants experienced substantial sleep disruption, with an average WASO of 65.52 ± 51.43 minutes and a mean snoring index of 210.57 ± 215.95 events per hour. The AHI was elevated, averaging 32.05 ± 26.08 events per hour. Sleep stage distribution indicated that N1 accounted for 11.27 ± 15.03% of TST, N2 for 28.99 ± 31.18%, N3 for 3.01 ± 6.78%, and REM for 7.28 ± 8.16%. The mean oxygen saturation (SpO_2_) was reported as 94.77 ± 2.30%. Body composition analysis revealed a FATP of −0.40 ± 1.86 and FATM of −0.04 ± 1.61. The predicted muscle mass (PMM) was 0.75 ± 1.27, and the VFATL was −0.08 ± 0.72. BONEM was 0.05 ± 0.09, while FFM was 0.79 ± 1.34. TBW, ECW, and ICW were 1.51 ± 1.58, 0.25 ± 0.40, and 1.26 ± 1.23, respectively. Metabolic indices showed a BMR of 98.18 ± 157.31 and a METAAGE of −0.67 ± 3.26. IMP was −36.22 ± 24.97, PHASEANGLE was −0.10 ± 0.75, and the PHYSRATE averaged 1.00 ± 4.67.

**Table 1:** Basic characteristics of study participants (N = 5,381).

Parameter	Mean ± SD
Age, years	47.17 ± 13.77
Male, % (n)	65.8 (3,531)
BMI, kg/m²	26.80 ± 5.10
Neck circumference, cm	37.56 ± 4.70
Waist circumference, cm	91.67 ± 12.99
Total sleep time, h	4.49 ± 1.03
Sleep efficiency, %	73.31 ± 16.64
WASO, min	65.52 ± 51.43
Snoring index, events/h	210.57 ± 215.95
AHI, events/h	32.05 ± 26.08
Sleep stages	
N1 (% of TST)	11.27 ± 15.03
N2 (% of TST)	28.99 ± 31.18
N3 (% of TST)	3.01 ± 6.78
REM (% of TST)	7.28 ± 8.16
Mean SpO_2_, %	94.77 ± 2.30
Body composition	
FATP	-0.40 ± 1.86
FATM	-0.04 ± 1.61
PMM	0.75 ± 1.27
VFATL	-0.08 ± 0.72
BONEM	0.05 ± 0.09
FFM	0.79 ± 1.34
TBW	1.51 ± 1.58
ECW	0.25 ± 0.40
ICW	1.26 ± 1.23
BMR	98.18 ± 157.31
METAAGE	-0.67 ± 3.26
IMP	-36.22 ± 24.97
PHASEANGLE	-0.10 ± 0.75
PHYSRATE	1.00 ± 4.67

AHI, apnea-hypopnea index; WASO, wake after sleep onset; TST, total sleep time; FATP, fat percentage; FATM, fat mass; PMM, predicted muscle mass; VFATL, visceral fat level; BONEM, bone mass; FFM, fat-free mass; TBW, total body water; ECW, extracellular water; ICW, intracellular water; BMR, basal metabolic rate; METAAGE, metabolic age; IMP, impedance; PHASEANGLE, phase angle; PHYSRATE, physical rating.

### 3.2 Associations between body composition and AHI

Linear regression analyses examining the associations between body composition, hydration status, and metabolic parameters with AHI are presented in **Table 2**. Unless otherwise specified, all predicators refer to overnight change (△) values. In Model 1, adjusted for age and sex, fat percentage (FATP: β = 1.27, 95% CI: 0.91 to1.63) and fat mass (FATM: β = 1.62, 95% CI: 1.20 to 2.03) were significantly positively associated with AHI (p < 0.05). Conversely, predicted muscle mass (PMM: β = −1.33, 95% CI: −1.87 to −0.80), visceral fat level (VFATL: β = −1.75, 95% CI: −2.67 to −0.82), bone mass (BONEM: β = −20.21, 95% CI: −27.72 to −12.69), and fat-free mass (FFM: β = −1.28, 95% CI: −1.78 to −0.77) showed significant negative associations with AHI (all p < 0.05). Among hydration indices, total body water (TBW: β = 1.17, 95% CI: 0.71 to 1.62) and intracellular water (ICW: β = 1.86, 95% CI: 1.27 to 2.44) were positively correlated with AHI, whereas extracellular water (ECW) was not significantly associated. Metabolic parameters such as basal metabolic rate (BMR: β = −0.01, 95% CI: −0.01 to −0.00) and physical rating (PHYSRATE: β = −0.31, 95% CI: −0.45 to −0.16) were inversely associated with AHI (p < 0.05), while metabolic age (METAAGE), impedance (IMP), and phase angle (PHASEANGLE) showed no significant associations. In Model 2, which additionally adjusted for BMI, the associations remained consistent or became more pronounced. Notably, the positive association between fat mass and AHI increased (β = 1.75, 95% CI: 1.23 to 2.28), while the inverse relationship between muscle mass and AHI strengthened (β = −1.73, 95% CI: −2.28 to −1.17). Similarly, stronger inverse associations were observed for VFATL (β = −3.94), BONEM (β = −25.05), and FFM (β = −1.65). Total body water and intracellular water remained significantly positively associated, while ECW continued to show no significant association.

**Table 2:** Associations of overnight change (△) in body composition, hydration, and metabolic parameters with AHI.

Variable	Model 1	Model 2
FATP	1.27 (0.91, 1.63)*	1.15 (0.77, 1.54)*
FATM	1.62 (1.20, 2.03)*	1.75 (1.23, 2.28)*
PMM	-1.33 (-1.87, -0.80)*	-1.73 (-2.28, -1.17)*
VFATL	-1.75 (-2.67, -0.82)*	-3.94 (-5.03, -2.84)*
BONEM	-20.21 (-27.72, -12.69)*	-25.05 (-32.75, -17.35)*
FFM	-1.28 (-1.78, -0.77)*	-1.65 (-2.17, -1.13)*
TBW	1.17 (0.71, 1.62)*	1.02 (0.56, 1.48)*
ECW	0.67 (-1.09, 2.43)	-0.84 (-2.74, 1.06)
ICW	1.86 (1.27, 2.44)*	1.74 (1.15, 2.32)*
BMR	-0.01 (-0.01, -0.00)*	-0.01 (-0.02, -0.01)*
METAAGE	0.13 (-0.08, 0.33)	0.03 (-0.18, 0.24)
IMP	-0.002 (-0.03, 0.03)	0.01 (-0.02, 0.03)
PHASEANGLE	-0.59 (-1.49, 0.30)	-0.63 (-1.52, 0.26)
PHYSRATE	-0.31 (-0.45, -0.16)*	-0.27 (-0.42, -0.13)*

Beta coefficients (β) with 95% confidence intervals (CI). Model 1 adjusted for sex and age. Model 2 adjusted for sex, age and BMI. *p<0.05.

### 3.3 Associations between physiological parameters, WASO, and sleep efficiency

As shown in **Table 3**, increased adiposity was significantly associated with longer wake after sleep onset (WASO). Specifically, BMI was positively associated with WASO (β = 4.07, 95% CI: 1.36 to 6.79), as were fat percentage (FATP: β = 1.17, 95% CI: 0.45 to 1.88), fat mass (FATM: β = 1.67, 95% CI: 0.84 to 2.50), and visceral fat level (VFATL: β = 2.72, 95% CI: 0.88 to 4.56). Conversely, bone mass was negatively associated with WASO (BONEM: β = −17.49, 95% CI: −32.46 to −2.52) and was inversely associated with WASO. For sleep efficiency, no adiposity or muscle-related metrics showed significant associations across efficiency categories (≥85%, 70–85%, ≤70%). However, basal metabolic rate (BMR) was significantly associated with lower sleep efficiency in the ≤70% group (β = 4.76, 95% CI: <0.001 to <0.001), and impedance (IMP) was also positively associated with poor sleep efficiency (85-70%) (β = 6.43, 95% CI: <0.001 to <0.001).

**Table 3:** Associations of overnight change (△) in body composition, hydration, and metabolic parameters with wake after sleep onset (WASO) and sleep efficiency.

Variable	WASO	Sleep efficiency			
		All	≧85%	85%-70%	≦70%
BMI	4.07(1.36, 6.79) *	0.70(-1.51, 0.65)	0.71(-1.58, 3.00)	0.00(-0.00, 0.01)	-0.00(-0.02, 0.01)
FATP	1.17(0.45, 1.88) *	-0.04(-0.20, 0.11)	0.17(-0.38, 0.73)	0.00(-0.00, 0.00)	-0.00(-0.00, 0.00)
FATM	1.67(0.84, 2.50) *	-0.05(-0.23, 0.12)	0.18(-0.50, 0.86)	-8.55(-0.00, 0.00)	-0.00(-0.01, 0.00)
PMM	-0.70(-1.77, 0.37)	0.12(-0.11, 0.35)	0.08(-0.73, 0.89)	0.00(-0.00, 0.00)	0.00(-0.00, 0.01)
VFATL	2.72(0.88, 4.56) *	-0.20(-0.59, 0.19)	-0.12(-1.54, 1.30)	0.00(-0.00, 0.00)	0.00(-0.01, 0.01)
BONEM	-17.49(-32.46, -2.52)	2.30(-0.87, 5.48)	1.99(-9.01, 12.99)	0.02(-0.01, 0.04)	0.00(-0.07, 0.07)
FFM	-0.70(-1.71, 0.31)	0.12(-0.10, 0.33)	0.08(-0.68, 0.84)	0.00(-0.00, 0.00)	0.00(-0.00, 0.01)
TBW	-0.32(-1.22, 0.58)	0.08(-0.11, 0.27)	-0.04(-0.71, 0.63)	0.00(-0.00, 0.00)	-0.00(-0.01, 0.00)
ECW	-1.43(-4.92, 2.06)	0.27(-0.47, 1.01)	0.11(-2.57, 2.79)	0.00(-0.00, 0.01)	0.00(-0.01, 0.02)
ICW	-0.37(-1.53, 0.79)	0.10(-0.15, 0.34)	-0.07(-0.93, 0.78)	0.00(-0.00, 0.00)	-0.00(-0.01, 0.00)
BMR	-0.00(-0.01, 0.01)	0.003 (-0.001, <0.001)	0.00(-0.01, 0.01)	4.76 (<0.001, <0.001)	4.33 (<0.001, <0.001)
METAAGE	0.82(0.41, 1.23) *	-0.04(-0.13, 0.05)	0.06(-0.23, 0.35)	0.00(-0.00, 0.00)	0.00(-0.00, 0.00)
IMP	-0.03(-0.08, 0.03)	0.00(-0.01, 0.01)	0.01(-0.03, 0.05)	6.43 (<0.001, <0.001)	0.00 (-0.001, <0.001) *
PHASEANGLE	-1.59(-3.37, 0.18)	0.19(-0.19, 0.56)	0.33(-1.10, 1.75)	0.00(-0.00, 0.00)	0.00(-0.00, 0.01)
PHYSRATE	-0.17(-0.46, 0.11)	-0.02(-0.08, 0.04)	-0.17(-0.38, 0.04)	4.21(0.00, 0.00)	0.00(-0.00, 0.00)

Beta coefficients (β) with 95% confidence intervals (CI). Models were adjusted for age and sex. *p<0.05; coefficients extremely close to zero are shown as < 0.001.

### 3.4 Associations between physiological parameters and sleep stage

**Table 4** presents the regression analysis of physiological parameters with sleep stage architecture. Fat mass (FATM) was positively associated with N1 sleep (β = 0.26, 95% CI: 0.01 to 0.51), while no significant associations were found between adiposity measures and N2, N3, or REM stages. Predicted muscle mass (PMM) showed a marginal association with increased N3 sleep and REM sleep. Bone mass (BONEM) was significantly associated with increased N2 (β = 9.62, 95% CI: 0.21 to 19.04), N3 (β = 2.10, 95% CI: 0.10 to 4.09), and REM sleep (β = 3.50, 95% CI: 1.03 to 5.97). Among hydration metrics, total body water (TBW) was positively associated with N1 (β = 0.53, 95% CI: 0.26 to 0.80), and intracellular water (ICW) was also significantly associated with N1 sleep (β = 0.75, 95% CI: 0.40 to 1.10). Extracellular water (ECW) was positively associated with REM sleep (β = 0.64, 95% CI: 0.06 to 1.22). Impedance (IMP) showed a significant inverse relationship with N2 sleep (β = −0.04, 95% CI: −0.07 to −0.01), while higher physical rating (PHYSRATE) was associated with reduced N1 sleep (β = −0.11, 95% CI: −0.19 to −0.02). Bone and muscle mass were positively associated with higher percentages of N2, N3 and REM, whereas higher impedance and lower physical rating were associated with a higher percentage of N1.

**Table 4:** Associations of overnight change (△) in body composition, hydration, and metabolic parameters with sleep stage .

Variable	N1	N2	N3	REM
BMI	0.98 (0.17, 1.80)	0.20 (-1.50, 1.91)	-0.00 (-0.37, 0.36)	0.19 (-0.26, 0.64)
FATP	0.17 (-0.05, 0.39)	-0.27 (-0.72, 0.18)	-0.09 (-0.18, 0.01)	-0.07 (-0.19, 0.05)
FATM	0.26 (0.01, 0.51)	-0.23 (-0.75, 0.29)	-0.09 (-0.20, 0.02)	-0.05 (-0.19, 0.09)
PMM	0.07 (-0.26, 0.39)	0.61 (-0.07, 1.28)	0.14 (-0.00, 0.28)	0.17 (-0.01, 0.34)
VFATL	-0.20 (-0.76, 0.35)	0.16 (-1.00, 1.31)	-0.03 (-0.27, 0.22)	-0.10 (-0.40, 0.21)
BONEM	0.06 (-4.46, 4.58)	9.62 (0.21, 19.04)	2.10 (0.10, 4.09)	3.50 (1.03, 5.97)
FFM	0.06 (-0.24, 0.36)	0.58 (-0.05, 1.22)	0.13 (-0.00, 0.27)	-0.16 (-0.00, 0.33)
TBW	0.53 (0.26, 0.80)*	0.37 (-0.19, 0.94)	-0.01 (-0.23, 0.02)	0.11 (-0.04, 0.26)
ECW	1.18 (0.13, 2.23)	1.31 (-0.88, 3.50)	0.17 (-0.30, 0.63)	0.64 (0.06, 1.22)
CW	0.75 (0.40, 1.10)*	0.47 (-0.25, 1.20)	-0.03 (-0.18, 0.13)	0.12 (-0.08, 0.31)
BMR	0.00 (-0.00, 0.00)	0.01 (-0.00, 0.01)	0.00 (-0.00, 0.00)	0.00 (0.00, 0.00)
METAAGE	0.03 (-0.09, 0.16)	-0.12 (-0.86, 0.14)	0.01 (-0.05, 0.06)	-0.04 (-0.11, 0.03)
IMP	-0.02 (-0.03, 0.00)	-0.04 (-0.07, -0.01)	0.00 (-0.01, 0.01)	-0.00 (-0.01, 0.01)
PHASEANGLE	0.07 (-0.47, 0.61)	0.39 (-0.73, 1.51)	0.18 (-0.06, 0.42)	0.26 (-0.03, 0.56)
PHYSRATE	-0.11 (-0.19, -0.02)	0.03 (-0.15, 0.21)	0.02 (-0.02, 0.06)	0.00 (-0.05, 0.05)

Beta coefficients (β) with 95% confidence intervals (CI). Models were adjusted for age and sex. * p <0.05.

### 3.5 Associations of sex- and BMI-specific body composition with AHI

**Table 5** presents the results of sex- and BMI-specific linear regression analyses assessing the associations between body composition, hydration, and metabolic parameters with AHI. In sex-stratified models (adjusted for age and BMI), fat percentage (FATP) and fat mass (FATM) were positively associated with AHI in both males (β = 0.94, 95% CI: 0.45 to 1.43; β = 1.27, 95% CI: 0.64 to 1.90, respectively) and females (β = 1.69, 95% CI: 1.07 to 2.31; β = 3.40, 95% CI: 2.43 to 4.36, respectively) (all p < 0.05). Conversely, muscle-related indices such as predicted muscle mass (PMM), fat-free mass (FFM), and bone mass (BONEM) showed significant negative associations with AHI, with stronger effects observed in females (e.g., PMM: β = −3.48, 95% CI: −4.51 to −2.45) than in males (β = −1.24, 95% CI: −1.91 to −0.58). Hydration parameters showed contrasting associations by sex: total body water (TBW), extracellular water (ECW), and intracellular water (ICW) were positively associated with AHI in males (e.g., TBW: β = 1.61, 95% CI: 1.08 to 2.13), but ECW negatively associated in females (β = −19.21, 95% CI: −22.82 to −15.59). Basal metabolic rate (BMR) was inversely associated with AHI in both sexes, with a stronger effect in females (β = −0.03, 95% CI: −0.04 to −0.02). Physical rating (PHYSRATE) also exhibited a significant negative correlation with AHI in both males and females (p < 0.05). In the BMI-stratified models (adjusted for age and sex), most associations between body composition and AHI were not statistically significant in the normal (BMI < 24) and overweight (BMI 24–27) subgroups. However, in the obese group (BMI ≥ 27), fat mass (β = 0.85, 95% CI: 0.26 to 1.44), muscle mass (β = −0.87, 95% CI: −1.72 to −0.02), bone mass (β = −15.16, 95% CI: −27.45 to −2.86), and total body water (β = 1.08, 95% CI: 0.39 to 1.77) showed significant associations with AHI (p < 0.05). In addition, intracellular water (ICW) was positively associated (β = 1.77, 95% CI: 0.87 to 2.67), while impedance (IMP: β = −0.08) was negatively associated with AHI in the obese subgroup (p < 0.05). In stratified models, the magnitude of associations between body composition, hydration status, and AHI differed by sex and BMI category, with stronger and more consistent relationships observed in females and individuals with obesity. For BMI categories, in the obese group, fat mass was significantly associated with increased AHI (FATM: β=0.85, CI: 0.26 to 1.44). Intracellular water (ICW) also showed a strong positive correlation (β=1.77, CI: 0.87 to 2.67), and total body water (TBW) was positively associated with AHI (β=1.08, CI: 0.39 to 1.77). Additionally, bone mass (BONEM) was negatively correlated with AHI (β=-15.16, CI: -27.45 to -2.86), consistent with higher AHI co-occurring with greater total and intracellular water in individuals with obesity.

**Table 5:** Associations of sex- and BMI-specific overnight change (△) in body composition, hydration, and metabolic parameters with the apnea-hypopnea index (AHI).

Variable	Sex		BMI		
	Male	Female	Normal (BMI < 24)	Overweight (24–27)	Obese (≥27)
FATP	0.94(0.45, 1.43)*	1.69(1.07, 2.31)*	0.13(-0.27, 0.53)	0.25(-0.35, 0.86)	0.71(0.08, 1.34)
FATM	1.27(0.64, 1.90)*	3.40(2.43, 4.36)*	0.20(-0.40, 0.80)	0.33(-0.49, 1.14)	0.85(0.26, 1.44)*
PMM	-1.24(-1.91, -0.58)*	-3.48(-4.51, -2.45)*	0.24(-0.45, 0.93)	-0.28(-1.15, 0.60)	-0.87(-1.72, -0.02)
VFATL	-4.41(-5.83, -2.98)*	-2.51(-4.16, -0.87)*	-0.54(-1.68, 0.60)	-0.18(-1.73, 1.37)	-1.68(-3.14, -0.21)
BONEM	-22.76(-33.56, -11.96)*	-29.45(-39.65, -19.24)*	-0.26(-9.25, 8.73)	-5.10(-17.79, 7.60)	-15.16(-27.45, -2.86)
FFM	-1.20(-1.83, -0.57)*	-3.18(-4.13, -2.23)*	0.21(-0.43, 0.86)	-0.27(-1.10, 0.56)	-0.84(-1.64, -0.04)
TBW	1.61(1.08, 2.13)*	-2.05(-3.09, -1.00)*	0.59(-0.02, 1.21)	0.33(-0.41, 1.07)	1.08(0.39, 1.77)*
ECW	4.08(1.85, 6.32)*	-19.21(-22.82, -15.59)*	1.77(-0.71, 4.24)	-0.62(-3.78, 2.55)	0.40(-2.15, 2.95)
ICW	2.25(1.58, 2.92)*	-0.82(-2.12, 0.48)	0.80(0.01, 1.59)	0.59(-0.35, 1.52)	1.77(0.87, 2.67)*
BMR	-0.01(-0.01, -0.00)*	-0.03(-0.04, -0.02)*	0.00(-0.00, 0.01)	-0.00(-0.01, 0.01)	-0.01(-0.01, 0.00)
METAAGE	-0.08(-0.33, 0.18)	0.38(-0.01, 0.76)	-0.14(-0.45, 0.17)	-0.03(-0.36, 0.31)	0.13(-0.18, 0.43)
IMP	0.01(-0.03, 0.05)	0.01(-0.03, 0.05)	-0.01(-0.04, 0.02)	-0.08(-0.12, -0.03)*	-0.08(-0.13, -0.03)*
PHASEANGLE	-0.13(-1.34, 1.07)	-1.60(-2.84, -0.35)	0.02(-1.16, 1.20)	-1.39(-2.69, -0.08)	-0.64(-2.11, 0.83)
PHYSRATE	-0.24(-0.42, -0.06)	-0.33(-0.57, -0.10)*	-0.03(-0.18, 0.12)	-0.08(-0.29, 0.13)	-0.13(-0.42, 0.16)

Beta coefficients (β) with 95% confidence intervals (CI). Sex models were adjusted for age and BMI. BMI models were adjusted for age and sex. *p <0.05.

### 3.6 Age-stratified analysis of physiological parameters

**Table 6** presents a comparative analysis of physiological parameters stratified by age (<50 vs. ≥50 years) with models adjusted for sex. Among participants aged <50 years, body mass index (BMI) (β = 4.03, 95% CI: 2.22 to 5.84), fat percentage (FATP: β = 1.59, 95% CI: 1.03 to 2.15), fat mass (FATM: β = 2.09, 95% CI: 1.49 to 2.69), and hydration markers (i.e. total body water (TBW: β = 1.74, 95% CI: 1.09 to 2.38), and intracellular water (ICW: β = 2.51, 95% CI: 1.68 to 3.33)) were all significantly elevated. In contrast, muscle mass (PMM: β =-1.45, 95% CI:-2.30 to -0.60), visceral fat (VFATL: β = −1.97, 95% CI: −3.31 to −0.63), bone mass (BONEM: β = −20.23, 95% CI: −31.90 to −8.55), and fat free mass (FFM: β =-1.38, 95% CI:-2.18 to -0.59) were significantly reduced in this group. Among participants aged ≥50 years, the associations were less pronounced; however, a significant increases were observed in FATP (β = 0.84, 95% CI: 0.39 to 1.30), FATM (β = 0.95, 95% CI: 0.38 to 1.51), TBW (β = 0.92, 95% CI: 0.30 to 1.53), and ICW (β = 1.60, 95% CI: 0.81 to 2.40). Bone mass was significantly lower in this group (BONEM: β = −17.22, 95% CI: −26.77 to −7.66), as was physical rating (PHYSRATE: β = −0.29, 95% CI: −1.48 to −0.11). These results indicate that while younger adults exhibited broad associations with AHI across adiposity, muscle, bone and hydration parameters, older adults showed significant associations mainly with fat mass, bone mass, hydration, and physical fitness.

**Table 6:** Associations of Age-Related Overnight Change (△) in Body Composition, Hydration, and Metabolic Parameters with the Apnea-Hypopnea Index (AHI).

Variable	Age < 50	Age ≥ 50
BMI	4.03 (2.22, 5.84)*	1.27 (-0.82, 3.35)
FATP	1.59 (1.03, 2.15)*	0.84 (0.39, 1.30)*
FATM	2.09 (1.49, 2.69)*	0.95 (0.38, 1.51)*
PMM	-1.45 (-2.30, -0.60)*	-0.89 (-1.56, 0.22)
VFATL	-1.97 (-3.31, -0.63)*	-1.42 (-2.68, -0.16)
BONEM	-20.23 (-31.90, -8.55)*	-17.22 (-26.77, -7.66)*
FFM	-1.38 (-2.18, -0.59)*	-0.87 (-1.50, -0.23)
TBW	1.74 (1.09, 2.38)*	0.92 (0.30, 1.53)*
ECW	3.11 (0.52, 5.70)	-0.46 (-2.79, 1.86)
ICW	2.51 (1.68, 3.33)*	1.60 (0.81, 2.40)*
BMR	-0.01 (-0.01, 0.00)	-0.01 (-0.01, 0.00)
METAAGE	0.21 (-0.10, 0.51)	0.05 (-0.23, 0.32)
IMP	0.00 (-0.04, 0.03)	-0.02 (-0.06, 0.02)
PHASEANGLE	-0.23 (-1.89, 1.43)	-0.74 (-1.76, 0.27)
PHYSRATE	-0.25 (-0.47, 0.02)	-0.29 (-0.48, -0.11)*

Beta coefficients (β) with 95% confidence intervals (CI). Models were adjusted for sex. * p < 0.05.

## 4 Discussion

The findings of this study show the multifactorial relationships between physiological parameters and sleep-disordered breathing, particularly OSA, as measured by the AHI. Notably, significant associations were observed between body composition, hydration status, and AHI, with both muscle and fat metrics playing critical roles. Increased fat mass and reduced muscle mass were significantly associated with higher AHI, aligning with previous evidence suggesting that excess adiposity is linked to greater airway collapsibility and inflammation, whereas lower lean mass may be associated with reduced airway stability (23–25). Recent studies corroborate associations of trunk fat and total body water with OSA severity and imaging work differentiates structural from functional muscle indices (26–28). Similarly, the positive associations between fat metrics and AHI emphasize the role of adiposity in exacerbating OSA severity (29), whereas the inverse relationships observed for muscle and bone mass highlight the potential protective effect of better musculoskeletal health, although the mechanisms remain complex (9). We observed bone mass showed a strong inverse relationship with AHI and WASO. Given that BIA-derived bone mass typically tracks overall physique greater lean and skeletal frame size with lower adiposity and fluid burden rather than a bone-specific effect. This interpretation is consistent with our boarder pattern, higher lean mass, and skeletal frame co-occurred with lower AHI and better sleep continuity, suggesting that the BONEM coefficient largely indexes overall physique rather than a bone-specific airway mechanism. Furthermore, the positive association between ICW and AHI suggests a potential role for fluid retention, may be related to airway narrowing during sleep (30).

In terms of sleep disruption, our findings demonstrated that increased WASO was significantly associated with higher BMI, fat percentage, visceral fat levels, and reduced bone mass. These results are consistent with previous research indicating that disrupted sleep is associated with greater adiposity and lower bone mineral density (31). In this cohort, WASO showed more frequent and stronger associations with body composition variables than sleep efficiency. This pattern highlights sleep continuity as a key phenotypic correlate; whether enhancing continuity improves metabolic or cardiometabolic outcomes requires prospective evaluation. This aligns with prior work showing that simple anthropometric parameters can rapidly and reliably identify cardiometabolic risk in patients with OSA without the need for laboratory tests (32).

When examining sleep stage architecture, we found that lighter sleep stages, particularly N1, were positively associated with BMI, TBW, and ICW, consistent with links between disrupted sleep and fluid retention. These findings align with existing literature indicating that poor sleep quality is associated with metabolic dysregulation, appetite hormone imbalance, and higher adiposity (33–36). In contrast, deeper sleep stages such as N2 and N3 were positively associated with muscle mass and bone indices; mechanistic implications require confirmation. Interestingly, VFATL did not show significant associations with sleep stage distribution, contrasting with some prior studies suggesting disrupted sleep increases visceral adiposity (37, 38). This discrepancy may be due to methodological differences, population variation, or the complexity of fat redistribution, highlighting the need for further mechanistic exploration. The association between REM sleep and bone mass also highlights potential systemic benefits of sleep architecture beyond cognitive function.

Sex- and BMI-stratified analyses further clarified these relationships. Stronger associations between AHI and body composition were observed in females and individuals with obesity, reinforcing findings from previous studies that link BMI with OSA severity (29, 39, 40). Our sex-stratified analyses revealed that hydration indices were positively associated with AHI in men but inversely or non-significantly associated in women, with a particularly strong negative association for ECW in women. This discrepancy may reflect hormonal influence on fluid regulation and airway physiology, as well as sex-specific difference in fat distribution. Women’s greater subcutaneous and peripheral fat depots may buffer extracellular fluid shifts differently from the visceral predominance in men, thereby modifying the effect of rostral fluid redistribution on pharyngeal tissues. Additional contributors, such as vascular compliance, lymphatic drainage or soft-tissue composition, may further account for these sex-specific patterns. In the obese group, increased fat mass and ICW were particularly associated with higher AHI, suggesting that both adiposity and fluid retention may contribute to sleep-disordered breathing (41, 42). These findings emphasize the significance of targeting both weight control and fluid regulation as part of comprehensive OSA management. From a clinical perspective, these subgroup findings suggest that weight control and fluid regulation should be core components of OSA management, especially in high-risk groups. Age-stratified analysis revealed that adiposity-related measures such as fat percentage, and fat mass remained significantly associated with AHI across both age groups, with stronger associations observed in individuals under 50 years. In contrast, older adults exhibited more pronounced reductions in bone mass and physical rating, consistent with aging-related physiological decline (43, 44). The shift from adiposity-driven factors in younger adults to a greater involvement of musculoskeletal and hydration factors in older adults, suggests a need for age-tailored preventive and therapeutic strategies (45–47).

Sex-specific trends were also evident. In younger males, obesity was associated with increased fat mass, TBW, ECW, and ICW, indicating a tendency toward both increased adiposity and fluid retention. In older males, body composition parameters were not significantly associated with AHI, with fat-related variables in particular showing a marked reduction in relevance with age. In contrast, adiposity remained a key factor across all age groups in females, with postmenopausal women particularly affected by increased visceral fat and reduced bone mass (48, 49). These observations support evidence that hormonal changes, particularly estrogen decline, may amplify OSA risk through metabolic and anatomical pathways.

A more granular, hierarchical analysis integrating age, sex, and BMI showed a reduction in predicted muscle mass and fat-free mass among younger obese women, although not statistically significant, suggesting a potential for early sarcopenic obesity (50, 51).

Moreover, ECW levels were significantly lower in younger obese individuals and trended similarly in older age groups, reflecting fluid distribution imbalances possibly related to inflammation and cellular water dysregulation (52, 53). In younger obese males, increased fat mass, TBW, ECW, and ICW were associated with OSA severity, supporting evidence that fluid intake and body composition interact in early adulthood (54–56). In older males, these associations diminished, suggesting a shift in the drivers of sleep-related outcomes from adiposity to lean mass and hydration stability (57). BMR, phase angle, and ECW showed limited associations across groups. Their clinical relevance may require larger datasets or more sensitive methodologies to detect meaningful effects. Nonetheless, these findings contribute to a broader understanding of physiological profiles linked to sleep health.

Our findings provide valuable guidance for tailoring interventions. Enhancing sleep quality, particularly deep sleep stages, may benefit muscle and bone health while reducing AHI. Weight loss, muscle preservation, and hydration management are crucial in younger and obese individuals, whereas older adults may benefit from interventions focused on lean mass retention and fluid balance. Such targeted strategies are essential for improving sleep-disordered breathing and related health outcomes. It is important to note that △ values derived from pre- to post-sleep BIA assessment may not exclusively represent true metabolic changes in fat or muscle compartments. BIA is highly sensitive to body fluid distribution, and an overnight recumbent position is known to induce a rostral fluid shift. Therefore, a proportion of the observed △ values may reflect postural fluid dynamics rather than actual alterations in tissue composition. This study has some limitations. Owing to its cross-sectional design, interpretation is limited to statistical associations between physiological parameters and sleep outcomes; predictive or causal claims are beyond the scope of this work. Furthermore, potential confounders such as lifestyle behavior, diet, physical activity, and comorbidities were not available in our study. In addition, there are more limitations that should be acknowledged. First as a retrospective single-center study, the generalizability of our findings may be limited, and selection bias cannot be excluded. Second, important biochemical markers such as lipid profiles, HbA1c, and inflammatory parameters were not available, which restricts our ability to explore mechanistic links with cardiometabolic risk. Third, we did not have an independent external validation cohort and replication in other populations will be needed to confirm our findings. Finally, although anthropometric and bioimpedance assessments were conducted by trained staff under standardized conditions, potential inter-observer variability cannot be entirely excluded.

## 5 Conclusion

In conclusion, this study identifies sleep quality, particularly the distribution of sleep stages, as a key factor associated with body composition and hydration status. These findings highlight the potential of targeting sleep architecture, especially enhancing deeper sleep and reducing lighter sleep stages, as a therapeutic approach to support metabolic and physiological health.

## Data Availability

The original contributions presented in the study are included in the article/supplementary material, further inquiries can be directed to the corresponding author/s.

## References

[1] Alterki A Abu-Farha M Al Shawaf E Al-Mulla F Abubaker J . Investigating the relationship between obstructive sleep apnoea, inflammation and cardio-metabolic diseases. Int J Mol Sci. (2023) 24:6807. doi: 10.3390/ijms24076807, PMID: 37047780 PMC10095553

[2] Carlone Baldino Garcia N Lopes WA Locateli JC Ferraz Simoes C de Oliveira GH de Souza Mendes VH . Multidisciplinary obesity treatment program improved health-related quality of life and positively correlated with anthropometric and body composition but not with cardiorespiratory fitness parameters in adolescents. Qual Life Res. (2019) 28:1803–12. doi: 10.1007/s11136-019-02141-9, PMID: 30790154

[3] Chen Y Li C Cheng S Pan C Zhang H Zhang J . The causal relationships between sleep-related phenotypes and body composition: A mendelian randomized study. J Clin Endocrinol Metab. (2022) 107:e3463–73. doi: 10.1210/clinem/dgac234, PMID: 35435981

[4] Uritani D Matsumoto D Asano Y Yoshizaki K Nishida Y Shima M . Effects of regular exercise and nutritional guidance on body composition, blood pressure, muscle strength and health-related quality of life in community-dwelling Japanese women. Obes Res Clin Pract. (2013) 7:e155–63. doi: 10.1016/j.orcp.2011.10.005, PMID: 24331777

[5] Kim SR Lee G Choi S Oh YH Son JS Park M . Changes in predicted lean body mass, appendicular skeletal muscle mass, and body fat mass and cardiovascular disease. J Cachexia Sarcopenia Muscle. (2022) 13:1113–23. doi: 10.1002/jcsm.12962, PMID: 35212175 PMC8978024

[6] Kuriyan R . Body composition techniques. Indian J Med Res. (2018) 148:648–58. doi: 10.4103/ijmr.IJMR_1777_18, PMID: 30666990 PMC6366261

[7] Tung NT Lin SY Dung HB Thuy TPC Kuan YC Tsai CY . Associations of overnight changes in body composition with positional obstructive sleep apnea. Sleep Breath. (2023) 27:631–40. doi: 10.1007/s11325-022-02664-5, PMID: 35752719

[8] Bonsignore MR . Obesity and obstructive sleep apnea. In: Eckel J Clément K , editors. From Obesity to Diabetes. Springer International Publishing, Cham (2022). p. 181–201. 10.1007/164_2021_558 34697666

[9] Matsumoto T Tanizawa K Tachikawa R Murase K Minami T Inouchi M . Associations of obstructive sleep apnea with truncal skeletal muscle mass and density. Sci Rep. (2018) 8:6550. doi: 10.1038/s41598-018-24750-z, PMID: 29695811 PMC5916913

[10] Drager LF Togeiro SM Polotsky VY Lorenzi-Filho G . Obstructive sleep apnea: a cardiometabolic risk in obesity and the metabolic syndrome. J Am Coll Cardiol. (2013) 62:569–76. doi: 10.1016/j.jacc.2013.05.045, PMID: 23770180 PMC4461232

[11] Ryan S Arnaud C Fitzpatrick SF Gaucher J Tamisier R Pepin JL . Adipose tissue as a key player in obstructive sleep apnoea. Eur Respir Rev. (2019) 28:190006. doi: 10.1183/16000617.0006-2019, PMID: 31243096 PMC9488701

[12] Balakrishnan G Burli D Behbehani K Burk JR Lucas EA . “ Comparison of a sleep quality index between normal and obstructive sleep apnea patients,” In: Proceedings of the 2005 IEEE Engineering in Medicine and Biology 27th Annual Conference, 17–18 Jan 2006. New York, NY, USA: IEEE. (2006). pp. 1154-7., PMID: 10.1109/IEMBS.2005.1616627 17282396

[13] Acquavella J Mehra R Bron M Suomi JM Hess GP . Prevalence of narcolepsy and other sleep disorders and frequency of diagnostic tests from 2013–2016 in insured patients actively seeking care. J Clin Sleep Med. (2020) 16:1255–63. doi: 10.5664/jcsm.8482, PMID: 32807293 PMC7446073

[14] Li CH Huang KY Chen WC Chen CH Tu CY Lin CL . Sleep disorders in individuals without sleep apnea increase the risk of peripheral arterial disorder: a nationwide population-based retrospective cohort study. Sleep Med. (2015) 16:966–70. doi: 10.1016/j.sleep.2015.02.538, PMID: 26143166

[15] Ohayon MM . Epidemiology of insomnia: what we know and what we still need to learn. Sleep Med Rev. (2002) 6:97–111. doi: 10.1053/smrv.2002.0186, PMID: 12531146

[16] Tiseo C Vacca A Felbush A Filimonova T Gai A Glazyrina T . Migraine and sleep disorders: a systematic review. J Headache Pain. (2020) 21:126. doi: 10.1186/s10194-020-01192-5, PMID: 33109076 PMC7590682

[17] Chang YH Chen YC Ku LE Chou YT Chen HY Su HC . Association between sleep health and intrinsic capacity among older adults in Taiwan. Sleep Med. (2023) 109:98–103. doi: 10.1016/j.sleep.2023.06.016, PMID: 37423025

[18] Chen CM Kuo CY Wu MN Hung JY Hsu CY Tsai MJ . Increased risk of major depressive disorder in sleep apnea patients in Taiwan. Sci Rep. (2021) 11:765. doi: 10.1038/s41598-020-80759-3, PMID: 33436925 PMC7803988

[19] Lin CE Chung CH Chen LF Chien WC Chou PH . The impact of antidepressants on the risk of developing obstructive sleep apnea in posttraumatic stress disorder: A nationwide cohort study in Taiwan. J Clin Sleep Med. (2019) 15:1233–41. doi: 10.5664/jcsm.7910, PMID: 31538594 PMC6760393

[20] Berry RB Brooks R Gamaldo C Harding SM Lloyd RM Quan SF . AASM scoring manual updates for 2017 (Version 2.4). J Clin Sleep Med. (2017) 13:665–6. doi: 10.5664/jcsm.6576, PMID: 28416048 PMC5406946

[21] Kapur VK Auckley DH Chowdhuri S Kuhlmann DC Mehra R Ramar K . Clinical practice guideline for diagnostic testing for adult obstructive sleep apnea: an american academy of sleep medicine clinical practice guideline. J Clin Sleep Med. (2017) 13:479–504. doi: 10.5664/jcsm.6506, PMID: 28162150 PMC5337595

[22] Tsai DH Riediker M Wuerzner G Maillard M Marques-Vidal P Paccaud F . Short-term increase in particulate matter blunts nocturnal blood pressure dipping and daytime urinary sodium excretion. Hypertension. (2012) 60:1061–9. doi: 10.1161/HYPERTENSIONAHA.112.195370, PMID: 22868388

[23] Ekingen T Sob C Hartmann C Ruhli FJ Matthes KL Staub K . Associations between hydration status, body composition, sociodemographic and lifestyle factors in the general population: a cross-sectional study. BMC Public Health. (2022) 22:900. doi: 10.1186/s12889-022-13280-z, PMID: 35513819 PMC9071243

[24] Laja Garcia AI Morais-Moreno C Samaniego-Vaesken ML Puga AM Varela-Moreiras G Partearroyo T . Association between hydration status and body composition in healthy adolescents from Spain. Nutrients. (2019) 11:2692. doi: 10.3390/nu11112692, PMID: 31703309 PMC6893474

[25] Serra-Prat M Lorenzo I Martinez J Palomera E Pleguezuelos E Ferrer P . Relationship between hydration status and muscle catabolism in the aged population: A cross-sectional study. Nutrients. (2023) 15:4718. doi: 10.3390/nu15224718, PMID: 38004111 PMC10674909

[26] Beyazal Polat H Ozyurt S Tastan M Beyazal Celiker F Beyazal M Sahin U . Muscle and fat composition in OSA: A CT-based study. J Clin Med. (2025) 14:4647. doi: 10.3390/jcm14134647, PMID: 40649021 PMC12250433

[27] Gao Y Zhao L Fan L Cai W Rui D Zhao Z . Decreased muscle percentage and increased fat percentage associated with obstructive sleep apnea syndrome severity in males: Insight from body composition and polysomnogram. Heliyon. (2025) 11:e143131. doi: 10.1016/j.heliyon.2025.e43131

[28] Yetim M Kalcik M Bekar L Karavelioglu Y Yilmaz YA . Body composition analysis in obstructive sleep apnea: A cross-sectional study using bioelectrical impedance analysis. Clin Respir J. (2025) 19:e70123. doi: 10.1111/crj.70123, PMID: 40968498 PMC12446078

[29] Bonsignore MR McNicholas WT Montserrat JM Eckel J . Adipose tissue in obesity and obstructive sleep apnoea. Eur Respir J. (2012) 39:746–67. doi: 10.1183/09031936.00047010, PMID: 21920888

[30] Liao Z Chen Y Wu L Huang Y Li S Liu J . Associations of obstructive sleep apnea risk with obesity, body composition and metabolic abnormalities in school-aged children and adolescents. Nutrients. (2024) 16:2419. doi: 10.3390/nu16152419, PMID: 39125300 PMC11313962

[31] Jurado-Fasoli L Amaro-Gahete FJ De-la OA Dote-Montero M Gutierrez A Castillo MJ . Association between sleep quality and body composition in sedentary middle-aged adults. Med (Kaunas). (2018) 54:91. doi: 10.3390/medicina54050091, PMID: 30463242 PMC6262283

[32] Zorlu D Boduroğlu Y Ertürk A . Echocardiographic evaluation from a different perspective in asthmatic patients. Tuberk Toraks. (2022) 70:166–78. doi: 10.5578/tt.20229807, PMID: 35785881

[33] Gangwisch JE . Epidemiological evidence for the links between sleep, circadian rhythms and metabolism. Obes Rev. (2009) 10 Suppl 2:37–45. doi: 10.1111/j.1467-789X.2009.00663.x, PMID: 19849800 PMC4075056

[34] Gonnissen HK Mazuy C Rutters F Martens EA Adam TC Westerterp-Plantenga MS . Sleep architecture when sleeping at an unusual circadian time and associations with insulin sensitivity. PloS One. (2013) 8:e72877. doi: 10.1371/journal.pone.0072877, PMID: 23951335 PMC3738551

[35] Gonnissen HK Rutters F Mazuy C Martens EA Adam TC Westerterp-Plantenga MS . Effect of a phase advance and phase delay of the 24-h cycle on energy metabolism, appetite, and related hormones. Am J Clin Nutr. (2012) 96:689–97. doi: 10.3945/ajcn.112.037192, PMID: 22914550

[36] Westerterp-Plantenga MS . Sleep, circadian rhythm and body weight: parallel developments. Proc Nutr Soc. (2016) 75:431–9. doi: 10.1017/S0029665116000227, PMID: 27117840

[37] Borel AL . Sleep apnea and sleep habits: relationships with metabolic syndrome. Nutrients. (2019) 11:2628. doi: 10.3390/nu11112628, PMID: 31684029 PMC6893600

[38] Nedeltcheva AV Scheer FA . Metabolic effects of sleep disruption, links to obesity and diabetes. Curr Opin Endocrinol Diabetes Obes. (2014) 21:293–8. doi: 10.1097/MED.0000000000000082, PMID: 24937041 PMC4370346

[39] Fattal D Hester S Wendt L . Body weight and obstructive sleep apnea: a mathematical relationship between body mass index and apnea-hypopnea index in veterans. J Clin Sleep Med. (2022) 18:2723–9. doi: 10.5664/jcsm.10190, PMID: 35929587 PMC9713905

[40] Leppanen T Kulkas A Mervaala E Toyras J . Increase in body mass index decreases duration of apneas and hypopneas in obstructive sleep apnea. Respir Care. (2019) 64:77–84. doi: 10.4187/respcare.06297, PMID: 30578359

[41] Hashiguchi MH Chubachi S Yamasawa W Otsuka K Harada N Miyao N . Interaction of BMI and respiratory status in obstructive sleep apnea, a cross-sectional COPD study. NPJ Prim Care Respir Med. (2023) 33:30. doi: 10.1038/s41533-023-00351-w, PMID: 37582926 PMC10427682

[42] Uzair A Waseem M Bin Shahid A Bhatti NI Arshad M Ishaq A . Correlation between body mass index and apnea-hypopnea index or nadir oxygen saturation levels in patients with obstructive sleep apnea. Cureus. (2024) 16:e59066. doi: 10.7759/cureus.59066, PMID: 38800192 PMC11128192

[43] Moreira VC Silva CMS Welker AF da Silva ICR . Visceral adipose tissue influence on health problem development and its relationship with serum biochemical parameters in middle-aged and older adults: A literature review. J Aging Res. (2022) 2022:8350527. doi: 10.1155/2022/8350527, PMID: 35492380 PMC9042620

[44] Briand M Raffin J Gonzalez-Bautista E Ritz P Abellan Van Kan G Pillard F . Body composition and aging: cross-sectional results from the INSPIRE study in people 20 to 93 years old. Geroscience. (2025) 47:863–75. doi: 10.1007/s11357-024-01245-6, PMID: 39028455 PMC11872965

[45] Palmer AK Jensen MD . Metabolic changes in aging humans: current evidence and therapeutic strategies. J Clin Invest. (2022) 132:e158451. doi: 10.1172/JCI158451, PMID: 35968789 PMC9374375

[46] Wang Y Shen S Han P Zheng K Chen C Wu Y . The association between visceral fat obesity and prefrailty in Chinese older adults: a cross-sectional study. BMC Endocr Disord. (2024) 24:136. doi: 10.1186/s12902-024-01625-1, PMID: 39090692 PMC11295587

[47] JafariNasabian P Inglis JE Reilly W Kelly OJ Ilich JZ . Aging human body: changes in bone, muscle and body fat with consequent changes in nutrient intake. J Endocrinol. (2017) 234:R37–51. doi: 10.1530/JOE-16-0603, PMID: 28442508

[48] Bonsignore MR Saaresranta T Riha RL . Sex differences in obstructive sleep apnoea. Eur Respir Rev. (2019) 28:190030. doi: 10.1183/16000617.0030-2019, PMID: 31694839 PMC9488655

[49] Wang Y Liu H Zhou B Yue W Wang M Hu K . Menopause and obstructive sleep apnea: revealing an independent mediating role of visceral fat beyond body mass index. BMC Endocr Disord. (2025) 25:21. doi: 10.1186/s12902-025-01850-2, PMID: 39863851 PMC11765922

[50] Palacios S Chedraui P Sanchez-Borrego R Coronado P Nappi RE . Obesity and menopause. Gynecol Endocrinol. (2024) 40:2312885. doi: 10.1080/09513590.2024.2312885, PMID: 38343134

[51] Woods R Hess R Biddington C Federico M . Association of lean body mass to menopausal symptoms: The Study of Women’s Health Across the Nation. Womens Midlife Health. (2020) 6:10. doi: 10.1186/s40695-020-00058-9, PMID: 32944260 PMC7490966

[52] van Marken Lichtenbelt WD Fogelholm M . Increased extracellular water compartment, relative to intracellular water compartment, after weight reduction. J Appl Physiol. (1999) 87:294–8. doi: 10.1152/jappl.1999.87.1.294, PMID: 10409587

[53] Mazariegos M Kral JG Wang J Waki M Heymsfield SB Pierson RN . Body composition and surgical treatment of obesity: effects of weight loss on fluid distribution. Ann Surg. (1992) 216:69–73. doi: 10.1097/00000658-199207000-00010, PMID: 1632704 PMC1242548

[54] Bossingham MJ Carnell NS Campbell WW . Water balance, hydration status, and fat-free mass hydration in younger and older adults. Am J Clin Nutr. (2005) 81:1342–50. doi: 10.1093/ajcn/81.6.1342, PMID: 15941885 PMC2495085

[55] Laja Garcia AI Morais-Moreno C Samaniego-Vaesken ML Puga AM Partearroyo T Varela-Moreiras G . Influence of water intake and balance on body composition in healthy young adults from Spain. Nutrients. (2019) 11:1923. doi: 10.3390/nu11081923, PMID: 31443311 PMC6723835

[56] Laja-García A de Lourdes Samaniego-Vaesken M Moráis-Moreno C Partearroyo T Varela-Moreiras G . The association between fluid intake, water balance and body composition. Proc Nutr Soc. (2020) 79:E655. doi: 10.1017/S0029665120006047

[57] Ramel ASS . Obesity and health in older adults. In: Geirsdóttir ÓGBJ , editor. Interdisciplinary Nutritional Management and Care for Older Adults: An Evidence-Based Practical Guide for Nurses. Springer, Cham (2021). p. 207–14.

